# Factors associated with utilization of LLINs among women of child-bearing age in Igabi, Kaduna State, Nigeria

**DOI:** 10.1186/s12936-019-3046-x

**Published:** 2019-12-10

**Authors:** Obafemi J. Babalola, Mohammed N. Sambo, Suleiman H. Idris, Ike-Oluwapo O. Ajayi, Olufemi Ajumobi, Patrick Nguku

**Affiliations:** 1 Liberia Field Epidemiology and Laboratory Training Programme, National Public Health Institute, Congo Town, Montserrado, Liberia; 2 Nigeria Field Epidemiology and Laboratory Training Programme (NFELTP), Asokoro District, Abuja, Nigeria; 3 Department of Community Medicine, Ahmadu Bello University, Zaria, Kaduna Nigeria; 4 Department of Epidemiology and Medical Statistics, University of Ibadan, Ibadan, Oyo Nigeria; 5 National Malaria Programme, Federal Ministry of Health, Abuja, Nigeria

**Keywords:** Malaria, Insecticide-treated bed nets, Ownership, Awareness, Knowledge, Nigeria

## Abstract

**Background:**

The long-lasting insecticidal nets (LLIN) are effective against prevention of malaria and its utilization has been proven to save lives. Despite the mass distribution of LLIN, Nigeria remains the country with the highest malaria burden in Africa. The awareness of LLIN in Nigeria is high, but the utilization is low. The aim of this work is to describe factors associated with the utilization of LLIN among women of child-bearing age (WCBA) in Igabi, Kaduna, Nigeria.

**Methods:**

A cross-sectional survey was conducted among 630 WCBA selected using a multi-stage sampling at 63 randomly selected villages in Igabi Local Government Area of Kaduna State. Trained female data collectors administered pre-tested structured questionnaires adapted from the Malaria Indicator Survey. Information collected were demographic profile, knowledge of LLIN as a preventive strategy for malaria, and LLIN ownership and utilization. LLIN utilization was assessed by identifying household members that slept under the hanged LLIN the night before the survey. Questions on the awareness of LLIN, ability to define what it is, use of LLIN, what differentiates LLIN from other bed nets, and duration of use before replacement, were scored and categorized as good, average and poor knowledge of LLIN.

**Results:**

A total of 629 WCBA was sampled, their mean age (± SD) was 29.3 (± 6.2) years, 22.0% were pregnant, 40.5% had no formal education, 41.1% were employed, and 47.7% lived in rural communities. Awareness and good knowledge about LLINs for the prevention of malaria was 96.0% and 24.0%, respectively. The proportion of women who slept under a LLIN the night before the survey (utilization) was 70.0% and slightly higher (74.0%) among pregnant WCBA. Women who lived in rural communities were more likely to utilize LLINs compared to their urban counterparts (OR 3.4; 95% CI 2.3–4.9). Younger women (aged < 30 years) were less likely to utilize LLINs compared to the older women (OR 0.7; 95% CI 0.5–0.9).

**Conclusions:**

The knowledge of LLIN among WCBA was poor, but LLIN utilization was moderate. Living in rural communities and older WCBA were significant characteristics associated with LLIN utilization. Strategies that will improve the utilization of LLIN among the young and urban WCBA should be the focus of the Malaria Elimination Programme (MEP).

## Background

Malaria is a global health concern with an estimated 219 million cases in 2017 and 92% of all cases from the Afro-region of the World Health Organization (WHO) [1]. Nigeria leads other ten African countries with a high burden of malaria and accounted for 25% of total malaria cases and 19% of malaria deaths worldwide in 2017 [1]. The use of long-lasting insecticidal nets (LLINs) is one of the integrated vector control strategies adopted on the Abuja Roll Back Malaria Initiatives Programme and has been proven to be a cost-effective way of saving lives [2, 3]. LLINs effectively averted 69% of 663 million malaria cases in 2015 [4]. LLIN household ownership in Nigeria increased from 44% in 2010 to 69% in 2015 [5, 6]. This was the result of routine and periodic mass LLIN distribution for rapid scale-up of LLINs in the communities. However, household LLIN ownership does not translate to utilization.

Factors affecting the utilization of LLIN among those that own at least one LLIN are numerous. Some of these factors are the perception of physical discomfort due to heat while sleeping under LLINs [7], low mosquito density in the sleeping room [7], the inability to hang available nets following mass distribution possibly due to lack of skill or no space to hang the nets [8], inadequate or no educational campaigns following LLIN distribution [8, 9], having a family size of 4 or less [10], not having heard of insecticide-treated nets in last 6 months [10], preference for specific shapes and colour of the nets [11], number of nets hung in the household for use by household members [11], and individual educational status and knowledge on the benefits of the LLIN utilization [12]. Non-utilization of LLIN may contribute to the high burden of malaria and slow progression to malaria elimination [1].

The role of awareness or knowledge to improve utilization of health commodities demonstrated in various studies has been equivocal [9, 13]. However, improved utilization of LLIN among pregnant women was attributed to their knowledge of the benefits of LLIN utilization and the risk of having malaria in pregnancy. Risks such as anaemia, low birth weight, and abortion make pregnant women use LLIN [14]. The terms “awareness” and “knowledge” are mostly interchanged [15]. According to Trevethan, the knowledge domain is a continuum from general knowledge of awareness to a piece of detailed and specific knowledge [15]. Therefore, providing detailed behavioural change communication interventions and specific knowledge on LLIN, rather than general knowledge or awareness would further promote LLIN utilization [9, 16–18].

High awareness and good knowledge of LLIN exist in Nigeria, but low utilization below the 80% target for population coverage persists [19]. In a household with at least one LLIN, 62% of women slept under an LLIN the previous night [6]. Women of child-bearing age (WCBA) described as women in the age group 15–49 years, are more vulnerable to malaria with low LLIN utilization [17]. Studies on LLIN utilization are however more focused on pregnant women, due to their vulnerability to malaria-causing maternal anaemia, preterm delivery, and low birth weight, rather than on all women of child-bearing age. Generally, women of child-bearing age are more likely to share their sleeping space with children under five; hence their net usage patterns help to protect children under 5 years from malaria. The objective of this study is to describe the characteristics of WCBA associated with LLIN ownership and utilization. The ethical approval referenced MOH/ADM/744/VOL.1/326 was obtained from the Ethical Review Committee of Ministry of Health, Kaduna State, Nigeria.

## Methods

### Study area

Igabi Local Government Area (LGA) with headquarters at Turunku, is one of the 23 LGAs in Kaduna State Nigeria. It has a population of 557,624 and is sub-divided into five urban districts, which are, Afaka, Birnin-yero, Kwarau, Rigachikun and Rigasa, and seven rural districts namely; Fanshanu, Gwaraji, Igabi, Kerawa, Sabo-Birnin, Turunku, and Zangon-aya. The LGA is located 650 m above sea level, between latitude 10° 47′ 0″ N and longitude 7° 46′ 0″ E in the tropical Sahel to Sudan Savannah with annual rainfall varying from 1000 to 1500 mm and highest precipitation of 72% in August. The rainy season is usually from May to October. The annual mean temperature is 34 °C. This can rise to 41 °C at the peak of the dry season in April and drop to as low as 12 °C in January during severe harmattan. This climatic condition influences the breeding and survival of malaria mosquitoes, and transmissions occur throughout the year. The incidence [20] and prevalence of malaria cases among under-five children at Igabi LGA is 13.6% and 55%, respectively. Also, 92% of households in Kaduna State have at least one LLIN and 62% slept under LLIN the previous night [6]. This study was conducted in October 2015 at the end of the rainy season. Six months before this study, the LGA benefitted from a mass household LLIN distribution campaign conducted in Kaduna state [20].

### Study design

This study employed a cross-sectional design.

### Study population

Women of child-bearing age, 15 to 49 years (WCBA) who reside in the selected households in the community for 12 months or more without cognitive deficits, or any chronic and debilitating illness that hinders participation in an effective interview were the participants.

### Sample size determination

Using the formula, n = z^2^pq/d^2^ at 95% confidence interval (1.96), prevalence of WCBA in North-Western Nigeria who slept under any net a night before, 43% [21] with a confidence limit of ± 5%, and a non-response rate of 10%. To adjust the required sample size for cluster sampling design, a correction factor of 1.5 is used as a design effect, as a less heterogenous community in this district was assumed. Therefore, the calculated minimum sample size was 630.

### Sampling technique

A three-stage cluster sampling technique was used to select the participants. Ten wards were randomly selected from the Igabi LGA. This included six rural wards—Fanshanu, Gwaraji, Igabi, Sabo-Birnin, Turunku, Zangon-aya and four urban wards—Afaka, Birnin-yero, Rigachikun, and Rigasa. These were selected at first stage making 2638 villages or settlements and 161,241 households. For a cluster or village/settlement selection, the household enumeration data generated by WHO/UNICEF during micro-planning for the mass LLIN campaign in these settlements were used as the sampling frame. With a sample size of 630, to improve the validity and precision of estimates and considering a wide community representation (population variance), using a probability proportional to size, 63 villages or clusters were randomly selected with a cluster size of 10.

Using the list of the households in each village/settlement generated during microplanning for mass LLIN distribution, ten households were systematically selected from each village/settlement. After the community entry at the traditional leader’s house, the first selected household was located and thereafter every Kth household was recruited into the study until all the 10 households. In a household with multiple eligible WCBA, one was selected by the ballot method and recruited into the study. Any household without eligible participants is an ineligible household.

### Data collection tools and technique

Data was collected from September to October 2015, by ten trained female community health workers who speak English and Hausa languages fluently and reside within the district. They were supervised by five undergraduate medical students. The data collectors used a pretested, structured questionnaire adapted from the Malaria Indicator Survey [22, 23] and other literature [24, 25] to collect information from the respondents. Information collected from the respondents included their demographic profile, knowledge of LLIN as a malaria prevention strategy, LLIN ownership and utilization, and influencers of LLIN utilization. The utilization of LLIN was verified by asking if the LLIN was used and who among the household members had slept under a LLIN the night before the survey. The interviewers also inspected and documented the conditions of all nets in the household to determine if they were used, unused, torn, hung, new or old. The LLIN knowledge items were questions from the literature, Malaria Indicator Survey and Demographic Health Survey. The adapted questions were reviewed by two malaria subject matter experts for face and content validity. They focused on the item’s accuracy, relevance and clarity. However, face validity was assessed by five malaria researchers and 15 WCBA for relevance to the WCBA. Following the review and corrections, each correct response was scored [9] “1” and incorrect “0”. The aggregate score for each respondent was expressed as percentage. Knowledge was categorized using percentile scores of 75th percentile or more for good knowledge, average knowledge between 50th and 74th percentile, and poor knowledge as < 50th percentile.

### Study variables

The outcome or dependent variable for this study was LLIN utilization. LLIN ownership was defined as having at least one LLIN within the household that may be used for sleeping and utilization is to sleep inside hung LLIN a night before the survey. Independent and explanatory variables are respondents’ demographic characteristics, LLIN ownership, and knowledge items.

### Data processing and analysis

Data was entered and analysed using Epi-Info version 7 statistical software. Frequency and proportions for sociodemographic characteristics, knowledge of LLIN, ownership and LLIN utilization were calculated. Ownership was the proportion of WCBA living in a household with any net for sleeping; its denominator was the total number of households sampled. Utilization referred to the proportion of respondents that slept under LLIN a night before the survey. Therefore, the denominator is the number of WCBA with LLIN. The mean (± standard deviation) or median for continuous descriptive variables, were also calculated. Bivariate analysis using odd ratio was done to determine the association between categorical outcome and independent variables and any associated factors with p-value ≤ 0.2 selected for a multivariable logistic regression model with an upward loading approach to identify factors independently influencing LLIN ownership and LLIN utilization among WCBA. For all statistical analyses, the significance level was set at a p-value of < 0.05 and a 95% confidence interval.

## Results

### Respondent’s demographic characteristics

The response rate was 99.8%. Table 1 shows the sociodemographic characteristics of the respondents. A total of 629 WCBA interviewed, their mean age (± standard deviation) was 29.3 (± 6.2) years, about 22.0% were currently pregnant, 40.5% had no formal education, 41.1% were employed, 47.7% were living in rural communities, 57.4% were housewife and 84.3% were Hausa by tribe.

**Table 1:** Sociodemographic characteristics of WCBA, Igabi LGA, Kaduna Nigeria, 2015 (N = 629)

Characteristics of WCBA	Frequency (n)	Percentage (%)
Age group (years)		
20–24	112	17.8
25–29	204	32.4
30–34	184	29.3
35–39	82	13.0
40–44	47	7.5
Educational level		
None	255	40.5
Primary	144	22.9
Secondary	174	27.7
Tertiary	56	8.9
Tribe		
Hausa	530	84.3
Fulani	39	6.2
Yoruba	15	2.4
Igbo	9	1.4
Others	36	5.7
Occupation		
Housewife	361	57.4
Trader	111	17.7
Civil servant	60	9.5
Craft/artisan	54	8.6
Farmer	34	5.4
Student	9	1.4
Marital status		
Married	620	98.6
Divorced/separated	9	1.4
Place of residence		
Rural	300	47.7
Urban	329	52.3

### Knowledge of women of child-bearing age on LLIN and associated factors

LLIN awareness among the women was 96.3% and 36.9% WCBA were able to differentiates LLIN from other bed nets by its factory impregnated insecticide (Table 2). LLIN was described by 45.0% WCBA as a free net given by the government, 29.6% as nets with impregnated insecticide that last longer more than 3 years, kill and prevent mosquito bite, and 21.1% of respondents said it is used for sleeping. The replacement period for LLIN was said to be > 2 years by 44.4% WCBA. On the use of LLIN, 67.6% responded that LLIN can be used to prevent mosquito bite and 36.9% knew that factory impregnated insecticide differentiates LLIN from other household nets. Overall, 24.0% (151/629) WCBA had good knowledge of LLIN (Table 3).

**Table 2:** Respondents knowledge of Long-Lasting Insecticidal Nets, Igabi LGA, Kaduna, 2015 (n = 629)

LLIN knowledge items	Frequency (n = 629)	Percentage (%)
Aware of LLIN		
Yes	606	96.3
No	23	3.7
Duration of LLIN awareness (months)		
< 13	308	49.0
13–24	237	37.7
> 24	61	9.7
Not aware	23	3.6
What is LLIN		
Free net given by government	283	45.0
Insecticide impregnated lasting > 3 years	186	29.6
Net for sleeping	133	21.1
Don’t know	27	4.3
Uses of LLIN		
Sleeping under prevent malaria	349	55.5
For sleeping only	170	27.0
Prevent mosquito bite	76	12.1
To build cage and fence farm	11	1.7
No response	23	3.7
Differentiate LLIN from other nets		
Factory impregnated insecticide	232	36.9
Don’t know	371	59.0
No response	22	3.5
Nothing	4	0.6
LLIN replacement period (years)		
< 1	37	5.9
1–2	288	45.8
> 2	279	44.4
Don’t know	25	3.9

**Table 3:** Level of knowledge classification of LLIN among WCBA, Igabi LGA, Kaduna, 2015

Knowledge classification	Frequency	Percentage (%)
Good	151	24.0
Average	318	50.6
Poor	160	35.4
Total	629	100

### LLIN ownership and utilization

In overall, 68.6% (341/497) of WCBA got LLIN in the last 7 months and ownership of at least one LLIN for sleeping by WCBA was 79% (497/629). The mean (± SD) household residence per LLIN was 2.6 (± 1.7) not different from the household with pregnant WCBA (Tables 4 and 5). Despite having at least one LLIN for sleeping, 6% (30/497) did not hang the available LLIN because they have no place to hang it and 13.1% (65/497) hanged more than enough bed nets for the use of household members. But 2.4% (12/497) did not hang the available LLIN because they did not know how to do it.

**Table 4:** Indicators of LLIN ownership and utilization among WCBA per wards, Igabi LGA, Kaduna, 2015 (n = 629)

Ward	Number N	Mean household residence per LLIN, Mean (± SD)	Proportion that owns at least 1 LLIN, n (%)	Proportion that slept under LLIN, n (%)	Proportion that owns at least 1 LLIN that slept under LLIN, n (%)
Among all women of child-bearing age					
Afaka	50	1.7 (0.6)	41 (82)	30 (60)	25 (61)
Birnin yero	40	2.0 (0.9)	32 (80)	30 (75)	24 (75)
Fanshanu	30	2.2 (1.1)	27 (90)	26 (87)	24 (89)
Gwaraji	40	1.9 (0.6)	36 (90)	30 (75)	27 (75)
Igabi	30	3.2 (2.0)	30 (100)	29 (97)	29 (97)
Rigachikun	50	2.7 (0.7)	42 (84)	41 (82)	41 (98)
Rigasa	229	3.1 (2.1)	144 (63)	121 (53)	83 (58)
Sabo birnin	70	2.8 (1.5)	61 (87)	59 (84)	50 (82)
Turunku	60	2.0 (1.2)	56 (93)	51 (85)	48 (86)
Zangon-aya	30	3.7 (3.2)	28 (93)	23 (77)	22 (79)
Total	629	2.6 (1.7)	497 (79)	440 (70)	373 (75)

**Table 5:** Indicators of LLIN ownership and utilization among pregnant WCBA per wards, Igabi LGA, Kaduna, 2015 (n = 137)

Ward	Number, N	Mean household residence per LLIN, Mean (± SD)	Proportion that owns at least 1 LLIN, n (%)	Proportion that slept under LLIN, n (%)	Proportion that owns at least 1 LLIN that slept under LLIN, n (%)
Among the pregnant women of child-bearing age					
Afaka	11	1.5 (0.5)	8 (73)	5 (45)	3 (37)
Birnin yero	8	2.2 (1.1)	8 (100)	5 (62)	5 (63)
Fanshanu	6	2.8 (0.9)	4 (67)	5 (83)	4 (100)
Gwaraji	9	1.6 (0.6)	9 (100)	7 (78)	7 (78)
Igabi	13	3.3 (1.1)	13 (100)	13 (100)	13 (100)
Rigachikun	1	2.0 (0.0)	1 (100)	1 (100)	1 (100)
Rigasa	44	2.6 (1.5)	28 (64)	27 (61)	19 (68)
Sabo birnin	16	3.6 (1.7)	14 (87)	14 (88)	12 (86)
Turunku	17	1.6 (0.9)	17 (100)	14 (82)	14 (82)
Zangon-aya	12	4.5 (4.1)	11 (92)	11 (92)	11 (100)
Total	137	2.6 (1.8)	113 (82)	102 (74)	89 (79)

Generally, LLIN utilization was 69.9% (440/629), but in a household with at least 1 LLIN for sleeping, 75% (373/497) WCBA utilized it. Among the 137 currently pregnant WCBA, 82% (112/137) owns at least one LLIN and 74% (101/137) utilized it. However, 79% (89/113) of pregnant WCBA with at least one LLIN slept under LLIN a night before.

The reasons for not having at least 1 LLIN by 132 WCBA were dislike to LLIN (56.8%), not knowing where to get LLIN (40.2%), using other malaria preventive measures (34.8%), no money to buy (25.8%), no voucher to collect free LLIN during mass distribution campaign (17.4%), stock out of LLIN during mass distribution campaign (8.3%), and loss the voucher during free LLIN mass distribution campaign (6.0%). Also, 11.5% (57/497) WCBA with at least one LLIN for sleeping in their household did not utilized it because of heat and discomforts (66.7%); poor perception that mosquitoes still bite while sleeping under LLIN (38.6%) and that no or low mosquito density in the sleeping room (22.8%); have a damaged or torn LLIN for sleeping (10.5%), LLIN in the household is inadequate as visitors used the LLIN (8.8%), and feeling of breathlessness or choking while sleeping under LLIN (7.0%).

### Factors influencing LLIN ownership

Table 6 shows the characteristic of WCBA associated with LLIN ownership. Women who lived in in rural communities were four times more likely to own LLINs compared to their urban counterpart (OR 3.8, CI 2.5–6.0, p < 0.01). Younger women (aged < 30 years) were less likely to own LLINs compared to the older women (OR 0.6, CI 0.4–0.6, p = 0.01). In Table 6, other respondents characteristics like having formal education (OR 0.6, CI 0.4–0.9, p = 0.03), and living in a household with less than three sleeping rooms (OR 0.4, CI 0.2–0.8, p = 0.01) were less likely factors associated with LLIN ownership.

**Table 6:** Respondents’ characteristic factors associated with LLIN ownership, Igabi LGA, Kaduna, 2015

Characteristics of WCBA	LLIN ownership (n = 629)		Odd ratio	95% CI
	Yes (n = 497)	No (n = 132)		
Age				
< 30 years	236 (74.7)	80 (25.3)	*0.6*	*0.4–0.9*
≥ 30 years	261 (83.4)	52 (16.6)	Ref	
Parity				
< 4	356 (79.5)	92 (20.5)	1.1	0.7–1.7
≥ 4	141 (77.9)	40 (22.1)	Ref	
Currently pregnant				
Yes	113 (82.5)	24 (17.5)	1.3	0.8–2.2
No	384 (78.0)	108 (22.0)	Ref	
Rural settlements				
Yes	269 (89.7)	31 (10.3)	*3.8*	*2.5–6.0*
No	228 (69.3)	101 (30.7)	Ref	
Had formal education				
Yes	284 (75.9)	90 (24.1)	*0.6*	*0.4–0.9*
No	213 (83.5)	42 (16.5)	Ref	
Good/average knowledge of LLIN				
Yes	423 (79.5)	109 (20.5)	1.21	0.7–2.0
No	74 (76.3)	23 (23.7)		
Available sleeping room(s) < 3				
Yes	392 (76.9)	118 (23.1)	*0.4*	*0.2–0.8*
No	105 (88.2)	14 (11.8)	Ref	
People slept here a night before < 5				
Yes	264 (80.2)	65 (19.8)	1.2	0.8–1.7
No	233 (77.7)	67 (22.3)	Ref	

Italics values indicate significance of p value (p < 0.05)

Women who are currently pregnant were more likely to own LLIN compared to other non-pregnant WCBA, but it was not significant (OR 1.3; CI 0.8–2.2, p = 0.3). The main predictor of LLIN ownership is rural residence as women who lived in rural communities were more likely to own LLINs when compare to the urban women (aOR 3.4; 95% CI 2.2–5.4).

### Factors influencing LLIN utilization

Generally, rural WCBA is strongly associated with LLIN utilization. Women who lived in rural communities were more likely to utilize LLINs than the urban women (OR 3.4 (95% CI 2.3–4.9) (Table 7). Also, younger women (aged < 30 years) were less likely to utilize LLINs compared to older women (OR 0.7; 95% CI 0.5–0.9, p = 0.03). Finally, after adjusting for these parameters age, currently pregnant, formal education and number of rooms for sleeping, women who lived in rural communities were more likely to utilize LLINs compared to their urban counterparts (aOR 3.9, CI 2.5–6.0, p < 0.01). Also, the number of rooms for sleeping in the households positively influences the utilization of LLINs among the respondents. Women who lived in a household with 2 or 3 rooms for sleeping were more likely to use LLIN compared to those living in 1 room (aOR 1.6, CI 1.1–2.4, p = 0.01).

**Table 7:** Respondents’ characteristic factors associated with LLIN utilization, Igabi LGA, Kaduna, 2015

Characteristics of WCBA	Utilization of LLIN		Odd ratio	95% CI
	Yes (n = 440)	No (n = 189)		
Age (years)				
< 30	208 (65.8)	108 (34.2)	*0.7*	*0.5*–*0.9*
≥ 30	232 (74.1)	81 (25.9)	Ref	
Parity				
< 4	315 (70.3)	133 (29.7)	1.1	0.7–1.5
≥ 4	125 (69.1)	56 (30.9)	Ref	
Currently pregnant				
Yes	103 (75.2)	34 (24.8)	1.4	0.9–2.1
No	337 (68.5)	155 (31.5)	Ref	
Rural settlements				
Yes	248 (82.7)	52 (27.3)	*3.4*	*2.3*–*4.9*
No	192 (58.4)	137 (41.6)	Ref	
WCBA had formal education				
Yes	251 (67.1)	123 (32.9)	0.7	0.5–1.0
No	189 (74.1)	66 (25.9)	Ref	
Good or average knowledge of LLIN				
Yes	377 (70.9)	155 (29.1)	1.31	0.8–2.1
No	63 (65.0)	32 (35.0)		
Available sleeping room(s) < 3				
Yes	350 (68.6)	160 (31.4)	0.7	0.4–1.1
No	90 (75.6)	29 (24.4)	Ref	
People slept here a night before < 5				
Yes	229 (69.6)	100 (30.4)	1	0.7–1.4
No	211 (70.3)	89 (29.7)	Ref	

Italics values indicate significance of p value (p < 0.05)

## Discussion

The study aimed at describing the characteristics of WCBA associated with LLIN ownership and utilization. The women population was younger (< 30 years of age) with the mean age of 29.3 (± 6.2) years and was not different from 28.8 (± 4.7) years found in a hospital-based study done among pregnant women in Abuja Nigeria [13]. Nearly half of the respondents lived in a rural community and every two out of five had no formal education. This reflects the literacy levels of WCBA in Kaduna State as reported in the Nigeria Demographic and Health Survey 2013 [5], although that of the general population of WCBA in Nigeria is slightly higher than 38.8% [6].

The general awareness of LLIN was high and similar to previous studies in Nigeria [19, 26]. The high general awareness of LLIN in this population may be due to previous interventions on communication and social mobilization for LLIN. However, the detailed and specific knowledge of LLIN was poor as only one out of four WCBA has good knowledge of LLIN [15, 27]. This study shows that a greater proportion of respondents with good knowledge have detailed knowledge of LLIN and its beneficial use in the prevention of malaria. The relationship between awareness and knowledge of LLIN and its utilization is equivocal. This was demonstrated in a study among pregnant women in Nigeria, where the knowledge that the use of insecticide treated nets prevents malaria strongly predicts its ownership [14]. It may be that these pregnant women received and understood a detailed and specific knowledge about LLIN and correctly linked the occurrence of malaria fever to the bites of infected mosquitoes; hence, the positive association between knowledge and LLIN ownership and utilization [28, 29]. However, in another study from eastern Nigeria, it was demonstrated that high awareness of LLIN does not relate to LLIN ownership and utilization [26].

Higher educational status and literacy levels have been adjudged to have an impact on the uptake of malaria preventive measures [13, 30, 31]. Most of the respondents reside in the urban community and the urban WCBA has better knowledge than rural women. Most urban women can easily be exposed to diverse sources of information on malaria and LLIN through the mass media and other means of public health communications. Also, they have more access to education than rural women. Although, the urban WCBA may have higher educational status [30] and exposure to communication materials on malaria and LLINs, but rural WCBA in this study owned and utilized LLIN more than the urban women. It may be that other factors rather than awareness, knowledge and educational status are responsible to LLIN ownership and utilization.

Overall, 79% WCBA owns at least 1 LLIN and 3 out of every 4 WCBA with LLIN ownership got it during the free mass LLIN campaign that was done 6 months earlier in the district. This could account for the high LLIN ownership among respondents as shown in a study from southwestern Nigeria where households’ LLIN ownership was linked to free mass LLIN distribution campaigns rather than from other sources [8]. Although the LLIN ownership in the study is lower than the 95% reported from a study from South Western Nigeria which was done following a free LLIN mass distribution campaign [8]. Also, it was lower than 91.6% reported for household LLIN ownership for Kaduna state in the 2015 Malaria Indicator Survey (MIS) [6]. Though LLIN ownership in this study was lower compared to the 91.6% reported in the MIS, this rate could have been an increase following free mass LLIN campaign. This further support the importance of this strategy to improve LLIN ownership and coverage [8, 32].

Furthermore, pregnant WCBA own more LLINs compared to non-pregnant WCBA. Due to the vulnerability of pregnant women, they are prioritized during free mass LLIN campaign and routine LLIN distribution at antenatal visits. More pregnant WCBA utilized LLIN than overall WCBA. The overall LLIN utilization was 70%, and 75% in WCBA living in a household with at least 1 LLIN. But among pregnant WCBA, LLIN utilization was 74%. This was higher than 67.8% reported for Kaduna State in 2015, MIS [6]. With this moderate increase, 80% of population coverage has not been reached and may accounted for high prevalence of malaria among this vulnerable group.

The main associated factor with LLIN ownership and utilization is rural residence. This positively influences LLIN ownership and utilization by WCBA. The finding was supported by other studies [6, 14, 33] from Nigeria. The high rural LLIN ownership by WCBA may be attributed to high rural community penetration of LLIN during free mass LLIN distribution campaigns [14]. Although, the urban women have high educational status, good awareness, knowledge of malaria and LLIN, but low LLIN utilization. This may be due to the availability, accessibility and the affordability of other malaria preventive measures in urban settings. This study also showed that, younger WCBA (age < 30 years) were less likely to own and utilize LLIN when compared to older women. This has been identified by another local study [34]. This may affect the drive for malaria prevention and elimination in Nigeria as this group constitutes a large part of the population, are caregivers of under-five children, and in early child-bearing age. Therefore, there is a need for further studies on why younger women averse to LLIN utilization.

LLIN ownership does not translate to utilization [14]. Therefore, barriers to LLIN ownership and utilization as reported in the literature exist among WCBA in Igabi, Kaduna Nigeria. These barriers could be responsible for the WCBA LLIN utilization gap as reported in this study. Some of these barriers include the unwillingness to own and use LLIN, not knowing where to get LLIN, using other malaria preventive measures, not having money to buy LLIN, not having a voucher to collect free LLIN during mass distribution campaign, and stock-out of LLIN during mass distribution campaigns. Other sources of LLIN utilization gap among WCBA living in a household with at least 1 LLIN but did not utilized it because of the following reasons: heat and discomfort, feeling of breathlessness or choking while sleeping under LLIN, perception that mosquitoes still bite while sleeping under LLIN, and that most sleeping rooms have low mosquito density. To avert these limitations, a detailed but more specific knowledge on the benefit of LLIN utilization among WCBA and their under-five children should be a focus of behavioural change communication to drive the closure of LLIN utilization gap.

### Limitations

The study was done at the peak of a malaria survey and about the same time when data collection for the 2015 Malaria Indicator Survey was ongoing. Nigeria is presently amongst the leading countries with a high burden of malaria and is making slow progress in efforts to reduce the malaria burden globally. The information on LLIN utilization was based on self-reported information from the participants. Data collectors observed the available LLIN in the household to know if they were hung, not hung, and if kept in its original packaging. The data collectors asked for who slept under a hung LLIN a night before the survey among the household members. Therefore, information bias like recall and social desirability are possible limitations. Efforts were made to reduce some of these limitations by piloting the questionnaire, training female data collectors to ensure standardized data collection processes, and participants were encouraged to be truthful with their responses and were assured of their confidentiality. However, it was not possible to verify actual LLIN utilization the previous night as well as confirm other self-reported data.

## Conclusion

The general awareness of LLIN among WCBA is good. The detailed and specific knowledge on LLIN is poor as one out of every four WCBA has good knowledge of LLIN. Although, the ownership and utilization of LLIN were moderate in all WCBA but higher among pregnant WCBA. Rural residents and older WCBA (30 years or more) were factors associated with LLIN ownership and utilization. Though the current awareness of LLIN is high, efforts should be made to redirect behavioural change communication from creating awareness to the dissemination of detailed and specific LLIN messages tailored toward building the WCBA’s knowledge on the benefit inherent in LLIN utilization [35]. The effect of malaria is more pronounced among pregnant women and WCBA was likely to share sleeping space with their under-five children. Hence, the net use patterns of WCBA extend to the protection of their under-five children and prevent malaria-causing morbidity and mortality during pregnancy. Therefore, strategies that will improve WCBA’s utilization of LLIN especially among the young and urban WCBA should be the focus of the Malaria Elimination Programme (MEP).

## Data Availability

The datasets used and/or analysed during the current study are available from the corresponding author on reasonable request.

## Abbreviations

LLIN: long-lasting insecticidal nets
LGA: Local Government Area
WCBA: women of child-bearing age
MIS: Malaria Indicator Survey
WHO: World Health Organization
UNICEF: United Nations Children’s Fund
